# Diagnosis of inborn errors of metabolism within the expanded newborn screening in the Madrid region

**DOI:** 10.1002/jmd2.12265

**Published:** 2022-01-27

**Authors:** Álvaro Martín‐Rivada, Laura Palomino Pérez, Pedro Ruiz‐Sala, Rosa Navarrete, Ana Cambra Conejero, Pilar Quijada Fraile, Ana Moráis López, Amaya Belanger‐Quintana, Elena Martín‐Hernández, Marcello Bellusci, Elvira Cañedo Villaroya, Silvia Chumillas Calzada, María Teresa García Silva, Ana Bergua Martínez, Sinziana Stanescu, Mercedes Martínez‐Pardo Casanova, Miguel L. F. Ruano, Magdalena Ugarte, Belén Pérez, Consuelo Pedrón‐Giner

**Affiliations:** ^1^ Sección de Gastroenterología y Nutrición Hospital Infantil Universitario Niño Jesús Madrid Spain; ^2^ Centro de Diagnóstico de Enfermedades Moleculares Universidad Autónoma de Madrid, IdiPAZ, CIBERER Madrid Spain; ^3^ Laboratorio de Cribado Neonatal de la Comunidad de Madrid Servicio de Bioquímica Clínica, Hospital General Universitario Gregorio Marañón Madrid Spain; ^4^ Unidad de Enfermedades Mitocondriales‐Metabólicas Hereditarias Centro de Referencia Nacional (CSUR) y Europeo (MetabERN) en Enfermedades Metabólicas, Hospital Universitario 12 de Octubre Madrid Spain; ^5^ Unidad de Nutrición Infantil y Enfermedades Metabólicas Hospital Universitario La Paz Madrid Spain; ^6^ Centro de Referencia Nacional (CSUR) en Enfermedades Metabólicas Hospital Universitario Ramón y Cajal Madrid Spain

**Keywords:** inborn error of metabolism, neonatal screening, tandem mass spectrometry

## Abstract

We present the results of our experience in the diagnosis of inborn errors of metabolism (IEM) since the Expanded Newborn Screening was implemented in our Region. Dried blood samples were collected 48 h after birth. Amino acids and acylcarnitines were quantitated by mass spectrometry (MS)/MS. Newborns with alterations were referred to the clinical centers for follow‐up. Biochemical and molecular genetic studies for confirmation of a disease were performed. In the period 2011 to 2019, 592 822 children were screened: 902 of them were referred for abnormal results. An IEM was confirmed in 222 (1/2670): aminoacidopathies: 89 hyperphenylalaninemia (HPA) (51 benign HPA, 32 phenylketonuria, 4 DNAJC12 defect, and 2 primapterinuria), 6 hypermethioninemia, 3 tyrosinemia type 1 (TYR‐1), 1 TYR‐3, 4 maple syrup urine disease (MSUD), 2 branched‐chain amino acid transferase 2 deficiency, 2 homocystinuria, 1 cystinuria, 2 ornithine transcarbamylase (OTC) deficiency, 2 citrullinemia type I (CTLN1); FAO defects: 43 medium‐chain acyl‐CoA dehydrogenase deficiency (MCADD), 13 very long‐chain acyl‐CoA dehydrogenase deficiency, 2 long‐chain 3‐hydroxyacyl‐CoA dehydrogenase deficiency (LCHADD), 1 multiple acyl‐coA dehydrogenation deficiency, 11 systemic primary carnitine deficiency, 2 carnitine palmitoyltransferase type 2 (CPT‐II) deficiency, 1 CPT‐I deficiency; organic acidurias: 12 glutaric aciduria type 1 (GA‐1), 4 methylmalonic acidemia (MMA), 7 MMA including combined cases with homocystinuria (MMAHC), 6 propionic acidemia (PA), 7 3‐methylcrotonyl‐CoA carboxylase, 1 3‐hydroxy‐3‐methylglutaryl‐CoA lyase deficiency lyase deficiency. Only 19 infants (8.5%) were symptomatic at newborn screening result (1 LCHADD, 5 PA, 1 CPT‐II deficiency, 1 MMA, 3 MMAHC, 2 MSUD, 2 OTC deficiency, 1 CTLN1, 1 MCADD, 2 TYR‐1). No false negative cases were identified. Genetic diagnosis was conclusive in all biochemically confirmed cases, except for two infants with HPA, identifying pathogenic variants in 32 different genes. The conditions with the highest incidence were HPA (1/6661) and MCAD deficiencies (1/13 787).

**Synopsis**

After the expanded newborn screening, 222 inborn errors of metabolism have been diagnosed in the Madrid Region, 203 of them in a presymptomatic phase.

## 1 INTRODUCTION

Newborn screening (NBS) started in the early 1960s with Robert Guthrie.^1^ In our country, the first NBS program was implemented in Granada in 1968 on the initiative of Prof Federico Mayor Zaragoza.^2^ Since then, programs have certainly improved as new technologies have evolved, especially after the introduction of tandem mass spectrometry (MS/MS) in the early 1990s.^3^, ^4^ MS/MS allows the simultaneous quantification of multiple analytes with high sensitivity and specificity, therefore it has been incorporated for inborn errors of metabolism (IEM) screening, providing the capability to detect over 50 conditions.^5^ However, countries have different approaches, and the situation varies considerably worldwide.^6^ In North America, a specific screening including which minimum IEM is established in all states.^7^ However, in the European Union, there is no agreement about which IEM should be screened.^8^ Furthermore, the protocols of the different countries vary in numerous aspects, from specimen collection to diagnosis, organization, follow‐up, and treatment.^9^ In Spain, as in other European Countries such as Belgium, Bosnia‐Herzegovina, Germany, Italy, or United Kingdom, the NBS program is carried out under the responsibility of different regions of the country.^10^ In 2013, the Spanish National Health System included, as mandatory, screening for seven genetic disorders, including four IEM (phenylketonuria [PKU], medium‐chain acyl‐CoA dehydrogenase deficiency [MCADD], long‐chain 3‐hydroxyacyl‐CoA dehydrogenase deficiency [LCHADD], and glutaric aciduria type 1 [GA‐1]).^11^ However, in practice, NBS is determined by the policies of individual regions, so there is a lack of uniformity in testing for disorders at birth around the country.^12^, ^13^ In the Community of Madrid, expanded NBS (ENBS) with MS/MS was initiated in 2011, including 17 metabolic disorders.^14^ In this article, the results of 9 years' experience in the diagnosis of IEM, after the implementation of the ENBS in our region, are described. Clinical and demographic data of the patients, along with the biochemical and the molecular characterization are presented.

## 2 PATIENTS AND METHODS

Metabolic disorders included in our ENBS program were: benign hyperphenylalaninemia (HPA)/ PKU, tyrosinemia type 1 (Tyr‐1), maple syrup urine disease (MSUD), MCADD, LCHADD, very long‐chain acyl‐CoA dehydrogenase deficiency, systemic primary carnitine deficiency (SPCD), 3‐hydroxy‐3‐methylglutaryl‐CoA lyase deficiency (HMG‐CoA lyase deficiency), GA‐1, methylmalonic acidemia (MMA) including combined cases with homocystinuria (MMAHC), propionic acidemia (PA), isovaleric acidemia, and beta‐ketothiolase deficiency. Furthermore, the different metabolites analyzed allows the identification of other IEM, such as homocystinuria, tyrosinemia type III, 3‐methylcrotonyl‐CoA carboxylase deficiency (MCG‐3), and some urea cycle defects, which are reported when detected, although not included “per se” in the program.

Dried blood samples were collected 48 h after birth and dried at room temperature. Amino acids, acylcarnitines, and succinylacetone were quantitated by NeoBase non‐derivatized MS/MS kit (PerkinElmer, Turku, Finland) from March 2011 to August 2018 and NeoBase 2 non‐derivatized MS/MS kit (PerkinElmer) from that moment to December 2019. Tandem mass spectrometer employed was an Acquity TDQ UPLC/MS (Waters, Milford, MA) system. For data acquisition and processing, the applications MassLynx (Waters), Neolynx and Specimen Gate MSMS Data Suite (PerkinElmer) were used. Our program analytes and ratios were selected following the Clinical and Laboratory Standards Institute recommendation.^15^ Cut‐off values were reviewed periodically. When the result for a newborn sample was flagged as above or under the cut‐off value (99.5th or 1st percentile), the analysis was repeated in duplicate before it was reported. If the repeat test was slightly outside the normal range, a second sample was requested by the NBS laboratory. On the other hand, if the result was clearly pathological, the patient was referred to one of the four reference clinical centers distributed by demographic criteria (Hospital Universitario 12 de Octubre, Hospital Universitario Ramón y Cajal, Hospital Universitario La Paz, Hospital Infantil Universitario Niño Jesús) for management, counseling, and follow‐up. Afterward, serum and urine samples were referred to the biochemical and molecular genetic laboratory (Centro de Diagnóstico de Enfermedades Moleculares), where they performed biochemical studies for confirmation of a disease, including plasma amino acids, acylcarnitines, urinary organic acid profiles, and molecular diagnosis thereafter to confirm the defect. Variants were classified following American College of Medical Genetics guidelines.^16^

In this study, we have included all the patients diagnosed with an IEM, who were referred to the clinical units for an abnormal screening between March 8, 2011 when ENBS was established in the Community of Madrid, and December 31, 2019.

Demographic and clinical conditions of the patients were registered, including sex, gestational age, presence or not of consanguinity, parents' country of origin, presence or not of symptoms at the time of the NBS result, biochemical and molecular diagnose, and if death occurred due to their pathology.

All these data were statistically analyzed with SPSS Statistics 24.

## 3 RESULTS

During the period included, 592.822 children were included in the ENBS program, 902 of them were referred due to an abnormal screening result. An IEM was confirmed in 222 cases (Table 1), which means an incidence of one case per 2.670 newborns (1/2670). Cases referred to the units without an IEM diagnosis are not included in this study and they correspond mostly to false positives cases, transitional alterations, heterozygote carriers, or vitamin B12 deficiency. The positive predictive value for the diagnosis of an IEM was 24.6%.

**TABLE 1:** Demographic, clinical, and biochemical data of patients diagnosed with an IEM by newborn screening in Madrid. Incidence is only shown for conditions included in the program

Biochemical diagnosis	MIM	Gene	GEN MIM	No. cases	Incidence	Days to clinical referral in asymptomatic patients mean (range)	Number of newborns with clinical symptoms before diagnosis (%)	Biochemical finding (median [range])
Disorders of amino acid metabolism								
Benign HPA	261 600	PAH	612 349	52	1/11 400	20.5 [7–117]	0	Phe: 172.8 μmol/L [97.5–244.2] Phe/Tyr: 2.1 [1.2–5.6]
Classic PKU	261 600	PAH	612 349	32	1/18 525	10.8 [5–41]	0	Phe: 576.8 μmol/L [225–1950] Phe/Tyr: 11.9 [1.8–32.2]
DNAJC12 deficiency	261 600	DNAJC12	606 060	4	NA	NA	0	Phe: 162.4 [123.1–265.0] Phe/Tyr: 1.8 [1.6–2.0]
Primapterinuria	264 070	PCBD1	126 090	2	1/296 411	12.5 [11–14]	0	Phe: 480, 193.4 μmol/L Phe/Tyr: 193.4, 3.6
GA1	231 670	GCDH	600 225	12	1/49 402	9.8 [5–30]	0	C5DC: 2.72 μmol/L [0.36–5.17]
MCG‐3‐MCC1D MCC2D	210 200 210 210	MCCC1 MCCC2	609 010 609 014	2 5	1/846 89	21.2 [7–58]	0	C5OH: 2.4 μmol/L [0.9–5.8]
PA	606 054	PCCB	232 050	6	1/98 804	9.5 [6–15]	5 (83%)	C3: 11.4 μmol/L [7.5–13.8] C3/C2: 0.9 [0.17–2.03] C3/Met: 0.9 [0.3–1.2]
MAT	250 850	MAT1A	610 550	6	NA	18.7 [9–41]	0	Met: 87.3 μmol/L [53–121]
TYR‐TYRSN1	276 700	FAH	613 871	3	1/197 607	6.7 [6–8]	1 (33.3%)	Tyr: 152.4 μmol/L [107.20–198] SA: 12.69 μmol/L [11.46–13.70]
TYRSN3	276 710	HPD	609 695	1	NA	13	0	Tyr: 558 mmol/L SA: 0.49 μmol/L
MSUD	248 600	BCKDHB	248 611	2	1/197 607	25.5 [8–43]	3 (75%)	Leu + Ile + Hyp: 1182 μmol/L [359–1911] Val: 375 μmol/L [137–616]
	248 600	BCKDHA	608 348	1				
		DBT	248 610	1				
BCAT‐2 deficiency	618 850	BCAT2	113 530	2	NA	11 [9–13]	0	Leu + Ile + Hyp: 298, 686 μmol/L Val: 312, 543 μmol/L
HC	236 200	CBS	613 381	2	NA	11.5 [10–13]	0	Met: 58.1, 92.0 μmol/L
Cystinuria	220 100	SLC3A1	104 614	1	NA	21	0	C3/C2: 0.24 μmol/L
OTC	311 250	OTC	300 461	1	NA	NA	1 (100%)	Cit: 2.5 μmol/L
CTLN1	215 700	ASS1	603 470	2	NA	11	0	Cit: 155, 1010 μmol/L
HMGCLD	246 450	HMGCL	613 898	1	NA	NA	0	C5OH: 0.8 μmol/L
Disorders of fatty acid oxidation and transport (FAO)								
MCADD	201 450	ACADM	607 008	43	1/13 787	11.3 [4–47]	1 (2.7%)	C8: 9.3 μmol/L [0.4–41.2] C8/C10: 10.4 [0.7–15.5]
VLCAD	201 475	ACADVL	609 575	13	1/45 602	12.4 [7–26]	0	C14:1:2.6 μmol/L [0.6–7.5]
LCHADD	609 016	HADHA	600 890	2	1/296 411	7	1 (50%)	C14:1:0.7, 0.7 μmol/L C16OH: 0.4, 0.8 μmol/L C18:OH: 0.5, 1.1 μmol/L
SPCD	212 140	SLC22A5	603 377	11	1/53 893	43.2 [8–128]	0	C0: 3.8 μmol/L [2.9–5.1]
CPT II	600 649	CPT2	600 650	2	NA	20	1 (50%)	C12: 0.6, 0,7 μmol/L C14: 1.6, 2.0 μmol/L C16:1:2.5, 2.7 μmol/L C18: 5.9, 5.7 μmol/L
MADD	231 680	ETFB	130 410	1	1/592 822	5	0	C8: 0.6 μmol/L C12: 1.9 μmol/L C14:1:1.7 μmol/L C16:OH: 0.5 μmol/L
CPT I	255 120	CPT1A	600 528	1	NA	11	0	C0: 94.7 μmol/L C16‐C18/C0: 160.5
Disorders of cobalamin metabolism								
MMAHC	277 400	MMACHC	609 831	6	1/84 689	20.4 [6–54]	3 (43%)	C3: 6.8 μmol/L [2.8–9.4] C3/C2: 0.71 [0.22–1.83] C3/Met: 1.07 [0.24–2.21]
	277 410	MMADHC	611 935	1				
MMA	251 000	MMUT	609 058	2	1/148 205	30.7 [19–38]	1 (25%)	C3: 6.30 μmol/L [3.9–9.1] C3/C2: 0.34 [0.24–0.6] C3/Met: 0.39 [0.22–0.72]
	251 110	MMAB	607 568	2				

Abbreviations: BCAT‐2, branched‐chain amino acid transferase 2 deficiency; CPT‐I, carnitine palmitoyltransferase type 1 deficiency; CPT‐II, carnitine palmitoyltransferase type 2 deficiency; CTLN1, citrullinemia type 1; DNAJC12, hyperphenylalaninemia due to DNAJC12 defect; GA‐1, glutaric aciduria type 1; HC, homocystinuria; HMG‐CLD, 3‐hydroxy‐3‐methylglutaryl‐CoA lyase deficiency; HPA, hyperphenylalaninemia; Hyp, Hydroxyproline; Ile, Isoleucine; LCHADD, long‐chain 3‐hydroxyacyl‐CoA dehydrogenase deficiency; Leu, Leucine; MADD, multiple acyl‐coA dehydrogenation deficiency; MAT, hypermethioninemia; MCADD, medium‐chain acyl‐CoA dehydrogenase deficiency; MCG‐3:3‐methylcrotonyl‐CoA carboxylase deficiency; MMA, methylmalonic acidemia; MMAHC, methylmalonic acidemia with homocystinuria; MSUD, maple syrup urinary disease; NA, not applicable; OTC, ornithine transcarbamylase deficiency; PA, propionic acidemia; PKU, phenylketonuria; SPCD, systemic primary carnitine deficiencies; TYRSN‐1, tyrosinemia type I; TYRSN‐3, tyrosinemia type 3; VLCAD, very long‐chain acyl‐CoA dehydrogenase deficiency.

IEM diagnosis included in the NBS program were 89 HPA (51 benign HPA, 32 classic PKU, 4 DNAJC12 defects, and 2 primapterinuria), 3 Tyr‐1, 4 MSUD, 43 MCADD, 2 LCHADD, 13 VLCAD, 11 SPCD, 12 GA‐1, 4 MMA, 7 MMAHC (6 cblC, 1 cblD), 6 PA, and 1 HMG‐CoA lyase deficiency. No cases of isovaleric acidemia nor beta‐ketothiolase deficiency were detected. Other metabolic conditions detected were: 6 hypermethioninemia, 1 tyrosinemia type 3, 2 branched‐chain amino acid transferase 2 deficiencies (BCAT‐2), 2 homocystinuria (cystathionine beta‐synthase deficiencies), 1 cystinuria, 2 ornithine transcarbamylase (OTC) deficiencies, 2 citrullinemia type I (CTLN1), 1 multiple acyl‐coA dehydrogenation deficiency (MADD), 2 carnitine palmitoyltransferase type 2 (CPT‐II) deficiency, 1 CPT 1 deficiency (CPT‐I), and 7 MCG‐3 (Table 1).

Of the total number of cases, 122 neonates were girls (55%). Median time to consultation in the clinical centers was 11 days of life (interquartile range: 8–17), being different according to the different pathologies as shown in Table 1. Nineteen of the patients (8.2%) had consanguineous parents and homozygous variants.

Only 19 infants (8.5%) were symptomatic at the time of the NBS result (1 LCHADD, 5 PA, 1 CPT‐II, 1 MMA, 3 MMAHC, 2 MSUD, 2 OTC deficiency, 1 CTLN1, 1 MCADD, 2 TYR‐1), displaying different clinical presentations and evolutions (Table 2). Three patients died due to their metabolic disorder, all of them had been detected in a symptomatic phase. One patient with LCHADD and one with OTC deficiency died in the first year of life. The other patient suffered from PA, he died at 3.5 years from a metabolic decompensation. So far, false negative cases have not been identified.

**TABLE 2:** Summary of patients who displayed with clinical symptoms before the newborn screening results were available

Case	IEM	NBS biochemical markers	Genotype	Days of life presenting with symptoms	Health care provider contact prior to NBS result	Clinical manifestations at diagnosis	Biochemical abnormalities	Detoxification measures at diagnosis	Long‐term follow‐up. Comorbidities
1	LCHAD	C16:1:0.41 μmol/L	*HADHA*: c.453 + 1G > A (p.?)/c.453 + 1G > A (p.?)	1	Yes	Acute respiratory distress and poor perfusion of peripheral tissues (dilated cardiomyopathy)	Hypoglycemia, metabolic acidosis, elevated lactate (12.1 mmol/L), hyperammonemia (134 μmol/L), CPK elevation (7.130 U/L)	Glucose 10 mg/kg/min and bicarbonate infusion	Death at 6 months
2	MCADD	C8: 41.2 μmol/L C8/C10: 15.5	*ACADM* c.985A > G (p.Lys329Glu)/c.985A > G (p.Lys329Glu)	2	No	Hypotonia	Hypoglycemia	No	No clinical alterations or metabolic decompensations
3	CPT‐II	C12: 0.7 μmol/L C14: 2.1 μmol/L C16:1:2.7 μmol/L C18: 5.7 μmol/L	*CPTII* c.1547 T > C (p.Phe516Ser)/c.122_130del9 (p.Pro41_Met43del)	7	No	Acute encephalopathy and seizures	Hypoglycemia, hyperammonemia (583 μmol/L)	Glucose 10 mg/kg/min	Autism spectrum disorder
4	PA	C3: 11 μmol/L C3/C2: 1.1 C3/Met: 1.2	*PCCB* c.1218_1231del14ins12 (p.Gly407Argfs*14)/c.1218_1231del14ins12 (p.Gly407Argfs*14)	2	Yes	Somnolence	Hypoglycemia, metabolic acidosis, hyperammonemia (480 μmol/L)	Glucose 10 mg/kg/min, arginine, carglumic acid, and ammonia scavengers	Hypotonia, pyramidal syndrome with severe cognitive impairment and epilepsy. Episodes of acute encephalopathy in absence of metabolic decompensation. Death at the age of 3.5 years because of breathing difficulty
5	PA	C3: 12.2 μmol/L C3/C2: 0.17 C3/Met: 0.61	*PCCB* c.1218_1231del14ins12 (p.Gly407Argfs*14)/ c.1218_1231del14ins12 (p.Gly407Argfs*14)	8	No	Lethargy, hypotonia, and urinary *Escherichia coli* infection	Metabolic acidosis, ketosis, hyperammonemia (239 μmol/L), pancytopenia	Glucose 10 mg/kg/min, arginine, carglumic acid, and ammonia scavengers	Several episodes of metabolic decompensation Severe–moderate cognitive impairment Liver transplantation
6	PA	C3: 12.4 μmol/L C3/C2: 2.0 C3/Met: 1.0	*PCCB* c.1173dupT (p.Val392Cysfs*2)/c.1218_1231del14ins12 (p.Gly407Argfs*14)	5	Yes	Encephalopathy and vomiting	Anemia, hyperammonemia (805 μmol/L), metabolic acidosis	Hemodiafiltration, carglumic acid, ammonia scavengers, l‐arginine, cofactors.	Cognitive impairment
7	PA	C3: 13.8 μmol/L C3/C2: 1.09 C3/Met: 1.18	*PCCB* c.1218_1231del14ins12 (p.Gly407Argfs*14)/c.1218_1231del14ins12 (p.Gly407Argfs*14)	7	Yes	Somnolence, poor feeding	Metabolic acidosis, hyperammonemia (585 μmol/L), anemia, thrombocytopenia, neutropenia	Glucose 10 mg/kg/min, arginine, carglumic acid, and sodium phenylbutyrate	Cognitive impairment and epilepsy
8	PA	C3: 14.36 μmol/L C3/C2: 0.67 C3/Met: 0.79	*PCCB* c.1173dupT(p.Val392Cysfs*2)/c.1173dupT(p.Val392Cysfs*2)	2	Yes	Somnolence, breathing difficulty	Hypoglycemia, hyperammonemia 7(234 μmol/L)	Glucose 10 mg/kg/min, arginine, and carglumic acid	Liver transplantation
9	MMAHC	C3: 9.5 μmol/L C3/C2: 0.5 C3/Met: 1.6	*MMACHC* c.271dupA (p.Arg91Lysfs*14)/c.271dupA (p.Arg91Lysfs*14)	12	No	Encephalopathy and seizures	Neutropenia	Cobalamin, folinic acid, and betaine	Visual and cognitive impairment
10	MMAHC	C3:3.80 μmol/L C3/C2: 1.02 C3/Met: 0.65	*MMACHC* c.271dupA (p.Arg91Lysfs*14)/c.271dupA (p.Arg91Lysfs*14)	18	No	Somnolence, poor feeding	Hypoglycemia, anemia, thrombocytopenia	Glucose 10 mg/kg/min	Cognitive impairment, epilepsy and growth restriction
11	MMAHC	C3: 8.72 μmol/L C3/C2: 0.52 C3/Met: 1.83	*MMACHC* c.271dupA (p.Arg91Lysfs*14) /c.271dupA (p.Arg91Lysfs*14)	11	Yes	Cardiomyopathy, hypotonia, seizures, jaundice, eczema, and urinary *E. coli* infection	Metabolic acidosis	Glucose 10 mg/kg/min	Cognitive impairment, behavioral disorder
12	MMA	C3: 9.17 μmol/L C3/C2: 0.60 C3/Met: 0.72	*MMAB* c.662 T > G (p.Phe221Cys)/c.569G > A (p.Arg190His)	13	No	Mild drowsiness	Metabolic acidosis, elevated lactate (7 mmol/L)	Glucose 10 mg/kg/min	Several episodes of metabolic decompensation (acidosis) Normal neurodevelopment
13	MSUD	Leu: 1390 μmol/L Val: 616 μmol/L	*BCKDHB* c.508C > T (p.Arg170Cys)/c.595_596delAG (p.Pro200Ter)	8	Yes	Severe encephalopathy (coma), fever, seizures, and facial and perianal eczema	Metabolic acidosis and hypernatremia	Hemodiafiltration	Cognitive impairment and liver transplantation (March 2018)
14	MSUD	Leu: 1070 μmol/L Val: 492 μmol/L	*DBT* c.(51 + 1_52–1)_(175 + 1_176–1)/c.(51 + 1_52–1)_(175 + 1_176–1)	6	Yes	Somnolence, poor feeding	Ketonuria+++ Mild hyperammonemia (126 μmol/L)	Hemodiafiltration	High leucine levels, with few episodes of metabolic decompensation Central nervous system lesions
15	TRSN1	SA: 11.46 μmol/L Tyr: 107.2 μmol/L	*FAH* c.554‐1G > T (p.?)/c.554‐1G > T (p.?)	8	No	No	Acute hepatic failure: hypoglycemia, coagulopathy	No	Mild cognitive impairment, epilepsy, and attention deficit hyperactivity disorder
16	TRSN1	SA: 13.70 μmol/L Tyr: 152 μmol/L	*FAH* c.G233A (p.Trp78Ter)/c.554‐1G > T (p.?)	16	No	No	Acute hepatic failure: coagulopathy, thrombocytopenia	No	Asymptomatic
17	OTC	Cit: 2.5 μmol/L	*OTC* c.928G > A (p.Glu310Lys)	3	Yes	Poor general clinical condition, respiratory distress, vomiting, seizures	Hyperammonemia (2484 μmol/L), coagulopathy, hypertransaminasemia	Hemodiafiltration, carglumic acid ammonia scavengers, l‐arginine, cofactors.	Death in neonatal period
18	OTC	Cit: 1.4 μmol/L	*OTC* c.77G > A (p.Arg26Gln)	5	Yes	Somnolence, poor feeding, vomiting	Metabolic acidosis hyperammonemia (1505 μmol/L)	Hemodiafiltration, carglumic acid, and sodium phenylbutyrate	Severe–moderate cognitive impairment
19	CTLN1	Cit: 1010 μmol/L	*ASS1* p.Val69Ala (c.206 T > C), p.Glu270Gln (c.808G > C)/p.Arg157His (c.470G > A)	6	Yes	Somnolence, decreased urine output, breathing difficulty.	Coagulopathy, hyperammonemia (800 μmol/L)	Hemodiafiltration	Normal neurodevelopment

Abbreviations: CPT‐II, carnitine palmitoyltransferase type 2 deficiency; CTLN1, citrullinemia type 1; IEM, inborn error of metabolism; LCHADD, long‐chain 3‐hydroxyacyl‐CoA dehydrogenase deficiency; MCADD, medium‐chain acyl‐CoA dehydrogenase deficiency; MMA, methylmalonic acidemia; MMAHC, methylmalonic acidemia with homocystinuria; MSUD, maple syrup urinary disease; NBS, newborn screening; OTC, ornithine transcarbamylase deficiency; PA, propionic acidemia.

Genetic diagnosis was performed in all biochemically confirmed cases, being conclusive in all of them, except for two patients with HPA: in whom only a variant in one allele in the *PAH* gene was found and no variants in the *DNAJC12* gens were identified. The different genotypes together with the geographical origin of the patients' parents are shown in Table 3. Analyses have detected 27 novel variants (supplementary material, S1 and S2), 18 pathogenic or likely pathogenic; 8 of them are variants with uncertain significance and one is potentially benign. The mutational spectrum included 17 potential missense variants, 3 small exonic insertion/deletions and 7 of them located in intronic sequence, and likely affecting the splicing process.

**TABLE 3:** Genotype of patients diagnosed with an IEM by newborn screening in Madrid

IEM (No. cases)	Gene	Genotype	No. cases	Country of origin
Benign HPA (51)	PAH	c.1208C > T (p.Ala403Val)/c.1208C > T (p.Ala403Val)	4	Morocco
		c.60 + 5G > T /c.158G > A (p.Arg53His)	2	Spain
		c.527G > T (p.Arg176Leu)/c.527G > T (p.Arg176Leu)	2	Dominican Republic
		c.842C > T (p.Pro281leu)/c.1139C > T (p.Thr380Met)	2	Georgia. Spain
		c.632delC (p.Pro211Hisfs*130)/c.734 T > C (p.Val245Ala)	1	Spain
		c.261C > A (p.Ser87Arg)/c.527G > T (p.Arg176Leu)	1	Spain
		c.441 + 5G > T /c.688G > A (p.Val230Ile)	1	Spain
		c.1243G > A (p.Asp415Asn)/c.441 + 5G > T	1	Spain
		c.165 T > G (p.Phe55Leu)/c.842 + 4A > G	1	Romania
		c.842C > T (p.Pro281Leu)/c.898G > T (p.Ala300Ser)	2	Romania. Spain
		c.734 T > C (p. Val 245Ala)/c.1241A > G (p.Tyr414Cys)	1	Spain
		c.194 T > C (p.Ile65Thr)/c.158G > A (p.Arg53His)	1	Spain
		c.158G > A (p.Arg53His)/c.510‐2A > G	1	Morocco
		c.117C > G (p.Phe39Leu)/c.183C > G (p.Asn61Lys)	1	Spain
		c.1045 T > C (p.Ser349Pro)	1	Spain
		c.1243G > A (p.Asp415Asn)/c.158G > A (p.Arg53His)	1	Morocco
		c.688G > A (p.Val230Ile)/c.1241A > G (p.Tyr414Cys)	1	Spain
		c.1262 T > C (p.Ile421Thr)/c.116_118delTCT (p.Phe39del)	1	Spain
		c.261C > A (p.Ser87Arg)/c.754C > T (p.Arg252Trp)	1	Spain
		c.1045 T > C (p.Ser349Pro)/c.1139C > T (p.Thr380Met)	1	Spain
		c.898G > T (p.Ala300Ser)/c.1241A > G (p.Tyr414Cys)	1	Spain
		c.527G > T (p.Arg176Leu)/c.898G > T (p.Ala300Ser)	1	Spain
		c.688G > A (p.Val230Ile)/c.1357*2delTAAAG (p.Ter453_Ser454delinsPro)	1	Spain‐Great Britain
		c.165 T > G (p.Phe55Leu)/c.1066‐11G > A (p.Gln355_Tyr356ins3)	1	Paraguay
		c.506G > A (p.Arg169His)/c.842C > T (p.Pro281Leu)	1	Georgia
		c.444 + 5G > T/c.809G > A (p.Arg270Lys)	1	Spain
		c.194 T > C (p.Ile65Thr)/c.688G > A (p.Val230Ile)	1	Spain
		c.1066‐11G > A (p.Gln355_Tyr356ins3)/c.1259 G > T (Arg420Met)	1	Spain
		c.510‐2A > G/c.158G > A (p.Arg53His)	1	Morocco
		c.898G > T (p.Ala300Ser) /c.441 + 5G > T	1	Spain
		c.165 T > G (p.Phe55Leu)/c.194 T > C (p.Ile65Thr)	1	Spain
		c.827 T > C (p.Met276Thr)/c.1208C > T (p.Ala403Val)	1	Spain
		c.838G > A (p.Glu280Lys)/c.1208C > T (p.Ala403Val)	1	Spain
		c.592_613del22 (p.Tyr198Serfs*136)/	1	Spain
		c.116_118delTCT (p.Phe39del)/c.165 T > G (p.Phe55Leu)	1	Spain
		c.60 + 5G > T /c.529G > A (p.Val177Met)	1	Spain
		c.1241A > G (p.Tyr414Cys)/c.1139C > T (p.Thr380Met)	1	Spain
		c.194 T > C (p.Ile65Thr)/c.1315 + 1G > A	1	Spain
		c.782G > A (p.Arg261Gln)/c.194 T > C (p.Ile65Thr)	1	Spain
		c.1066‐11G > A (p.Gln355_Tyr356ins3)/c.805A > C (p.Ile269Leu)	1	Spain
		c.898G > T (p.Ala300Ser)/c.1065 + 3A > C	1	Spain
		c.1208C > T (p.A403V)/c.441 + 5G > T	1	Spain
		c.1066–11 G > A /c.1199 + 17 G > A	1	Spain
		c.746 T > C (p.Leu249Pro)/c.890G > A (p.Arg297His)	1	Spain
Classic PKU (32)	PAH	c.842C > T (p.Pro281leu)/c.1162G > A (p.Val388Met)	2	Spain
		c.754C > T (p.Arg252Trp)/c.1066‐11G > A (p.Gln355_Tyr356ins3)	1	Spain
		c.185_189delTGACC (Leu62Profs*3)/c.441 + 5G > T	1	Paraguay
		c.1243G > A (p.Asp415Asn)/c.442‐?_509 +?del (p.Gly148Trpfs*29?)	1	Colombia
		c.781C > T (p.Arg261Ter)/c.1223G > A (p.Arg408Gln)	1	Spain
		c.1222C > T (p.Arg408Trp)/c.1222C > T (p.Arg408Trp)	1	Romania
		c.143 T > C (p.Leu48Ser)/c.441 + 5G > T	1	Spain
		c.1027 T > G (p.Tyr343Asp)/c.1162G > A (p.Val388Met)	1	Spain
		c.1045 T > C (p.Ser349Pro)/c.506_508delGCCinsCCA, p.(Arg169_His170delinsProAsn)	1	Spain
		c.1055delG (p.Gly352Valfs*48)/c.1055delG (p.Gly352Valfs*48)	1	Morocco
		c.204A > T (p.Arg68Ser)/c.136G > A (p.Gly46Ser)	1	Cuba
		c.533A > G (p.Glu178Gly)/c.1222C > T (p.Arg408Trp)	1	Romania
		c.1241A > G (p.Tyr414Cys)/c.1315 + 1G > A	1	Spain
		c.165 T > G (p.Phe55Leu)/c.782G > A (p.Arg261Gln)	1	Ecuador/Cuba
		c.500A > T (p.Asn167Ile)/c.1223G > A (p.Arg408Gln)	1	Germany/Spain
		c.782G > A (p.Arg261Gln)/c.1162G > A (p.Val388Met)	1	Spain/Portugal
		c.782G > A (p.Arg261Gln)/c.842C > T (p.Pro281Leu)	1	Spain
		c.721C > T (p.Arg241Cys)/c.721C > T (p.Arg241Cys)	1	Morocco
		c.439C > T (p.Pro147Ser)/c.727C > T (p.Arg243Ter)	1	Spain
		c.441 + 5G > T /c.782G > A (p.Arg261Gln)	1	Spain
		c.754C > T (p.Arg252Trp)/c.782G > A (p.Arg261Gln)	1	Bulgaria
		c.60 + 5G > T /c.1055delG (p.Gly352Valfs*48)	1	Spain
		c.842C > T (p.Pro281leu)/c.842C > T (p.Pro281leu)	1	Morocco
		c.143 T > C (p.Leu48Ser)/c.1222C > T (p.Arg408Trp)	1	Romania
		c.441 + 5G > T /c.1066‐11G > A (p.Gln355_Tyr356ins3)	1	Spain
		c.1162G > A (p.Val388Met)/c.1162G > A (p.Val388Met)	1	Spain
		c.561G > C (p.Trp187Cys)/c.1241A > G (p.Tyr414Cys)	1	Peru/Spain
		c.441 + 5G > T /c.1028A > G (p.Tyr343Cys)	1	Spain
		c.781C > T (p.Arg261Ter)/c.1262 T > C (p.Ile421Thr)	1	Spain
		c.1067‐11G > A /c.1067‐11G > A	1	Morocco
		c.1241A > G (p.Tyr414Cys)/c.1042C > G (p.Leu348Val)	1	Spain
DNAJC12 deficiency (4)	DNAJC12	c.524G > A (p.Trp175Ter)/c.524G > A (p.Trp175Ter)	2	Spain
		c.524G > A (p.Trp175Ter)/c.502 + 1G > C	1	Spain
		c.524G > A (p.Trp175Ter)/c.298‐2A > C	1	Spain
Primapterinuria (2)	PCBD1	c.259G > T (p.Glu87Ter) /c.259G > T (p.Glu87Ter)	1	Cape Verde
		c.292C > T(p.Gln98Ter) /c.292C > T(p.Gln98Ter)	1	Spain
GA‐1 (12)	GCDH	c.1198G > A (p.Val400Met)/c.1198G > A (p.Val400Met)	2	Spain
		c.1198G > A (p.Val400Met)/c.1240C > T (p.Arg402Trp)	1	Spain
		c.1198G > A (p.Val400Met)/c.442G > T (p.Val148Phe)	1	Spain
		c.278A > G (p.His93Arg)/c.278A > G (p.His93Arg)	1	Spain
		c.877G > A (p.Ala293Thr)/c.877G > A (p.Ala293Thr)	1	Spain
		c.877G > A (p.Ala293Thr)/c.1198G > A (p.Val400Met)	1	Spain
		c.877G > A (p.Ala293Thr)/c.1210G > C (p.Ala404Pro)	1	Spain
		c.946G > A (p.Ala304Thr)/c.1198G > A (p.Val400Met)	1	Spain
		c.442G > T (p.Val148Phe)/c.463 T > C (p.Tyr1555His)	1	Spain
		c.395G > A (p.Arg132Gln)/c.1204C > T (p.Arg402Trp)	1	Spain
		c.1144G > A (p.Ala382Thr) /c.1204C > T (p.Arg402Trp)	1	Dominican Republic
MCG‐3 (7)	MCCC1	c.1331G > A (p.Arg444His) /c.1008G > C (p.Met336Ileu)	1	Morocco
		c.872 (p.Ala291Val) /c.1970 T > C (p.Ile657Thr)	1	Spain
	MCCC2	c.1015G > A (p.Val339Met)/c.1635dupT (p.Ser546Ter)	1	Spain
		c.1322 T > C (p.Ile441Thr)/c.129 + 3A > G	1	Spain
		c.1015G > A (p.Val339Met)/c.641G > C (p.Gly214Ala)	1	Spain
		1423G > A (p.Gly475Arg)/c.1423G > A (p.Gly475Arg)	1	Morocco
		c.804‐14 T > A /c.804‐14 T > A	1	Ecuador
PA (5)	PCCB	c.1218_1231del14ins12 (p.Gly407Argfs*14)/c.1218_1231del14ins12 (p.Gly407Argfs*14)	3	Spain (2). Spain‐Peru (1)
		c.1218_1231del14ins12 (p.Gly407Argfs*14) /c.1173dupT (p.Val392Cysfs*2)	1	Spain
		c.1606A > G (p.Asn536Asp) /c.1606A > G(p.Asn536Asp)	1	Spain
		c.1173dupT(p.Val392Cysfs*2) /c.1173dupT(p.Val392Cysfs*2)	1	Spain
MAT (6)	MAT1A	c.791G > A (p.Arg264His)	4	Spain (3). Argentina (1)
		c.776C > T (p.Ala259Val)	1	Spain
		c.595C > T (p.Arg199Cys) /c.770G > A (p.Gly257Glu)	1	Spain
TYRSN1 (3)	FAH	c.554‐1G > T /c.554‐1G > T	2	Spain/Morocco
		c.554‐1G > T /c.233G > A p.(Trp78Ter)	1	Spain
TYRSN3 (1)	HPD	c.778G > A (p.Gly260Arg)/c.1118A > T (p.Glu373Val)	1	Ecuador
MSUD (4)	BCKDHB	c.508C > T (p.Arg170Cys) /c.595_596delAG (p.Pro200Ter)	1	Spain
		c.595_596delAG (p.Pro200Ter) /c.604G > A (p.Ala202Thr)	1	Spain‐Colombia
	BCKDHA	c.370C > T (p.Arg124Trp)/c.743C > T (p.Ala248Val)	1	Paraguay
	DBT	c.(51 + 1_52–1)_(175 + 1_176–1)/c.(51 + 1_52–1)_(175 + 1_176–1)	1	El Salvador
BCAT‐2 deficiency (2)	BCAT2	c.1154_1160del7ins12 (p.Ala385Valfs*35)/c.1154_1160del7ins12 (p.Ala385Valfs*35)	1	Spain
		c.762G > C (p.Trp254Cys) /c.923G > A (p.Trp308Ter)	1	Spain
HC (2)	CBS	c.572C > T (p.Thr191Met) /c.572C > T (p.Thr191Met)	1	Spain
		c.770C > T (p.Thr257Met) /c.803 T > C (p.Leu268Pro)	1	Netherlands/Spain
Cystinuria (1)	SLC3A1	c.797 T > C (p.Phe266Ser)/c.1400 T > C (p.Met467Thr)	1	Romania
OTC (2)	OTC	c.928G > A (p.Glu310Lys)	1	Spain
		c.77G > A (p.Arg26Gln)		Venezuela
CTLN1 (2)	ASS1	c.[267 T > C;808G > C] (p.Val69Ala + Glu270Gln) /c.805G > A (p.Val269Met)	1	Spain/Peru
		c.[206 T > C;808G > C] (p.[Val69Ala;Glu270Gln]) /c.470G > A (p.Arg157His)	1	Spain
HMGCLD (1)	HMGCL	c.109G > T (p.Glu37Ter) /c.785G > A (p.Gly262Glu)	1	Spain/Argentina
MCADD (43)	ACADM	c.985A > G (p.Lys329Glu)/c.985A > G (p.Lys329Glu)	22	Spain (19). Romania (2) Peru (1)
		c.985A > G (p.Lys329Glu)/c.638C > A (p.Thr228Asn)	7	Spain
		c.638C > A (pThr228Asn)/c.999_1011dup13 (p.Glu338Ter)	2	Spain
		c.985A > G (p.Lys329Glu)/c.626C > T (p.Pro209Leu)	2	Spain
		c.985A > G (p.Lys329Glu)/c.351A > C (p.Thr117Thr)	1	Romania/Colombia
		c.985A > G (p.Lys329Glu)/c.799G > A (p.Gly267Arg)	1	Spain
		c.985A > G (p.Lys329Glu)/c.250C > T (p.Leu84phe)	1	Spain/Canada
		c.985A > G (p.Lys329Glu)/c.946‐2A > C	1	Spain
		c.985A > G (p.Lys329Glu)/c.609A > C (p.Leu203Phe)	1	Spain
		c.985A > G (p.Lys329Glu)/c.127G > A (p.Glu43Lys)	1	Spain
		c.985A > G (p.Lys329Glu)/c.599 + 3A > G	1	Spain/Paraguay
		c.351A > C (p.Thr117Thr)/c.503A > C (p.Asp168Ala)	1	Spain
		c.338C > A (p.Ala113Asp)/c.940G > C (p.Val314Leu)	1	Ecuador
		c.1247 T > C (p.Ile416Thr)/c.778_782delGAAAA (p.Glu260Cysfs*5)	1	Paraguay
VLCAD (13)	ACADVL	c.848 T > C (p.Val283Ala)/c.1220G > C (p.Gly407Ala)	2	Spain
		c.848 T > C (p.Val283Ala)/c.685 > T (p.Arg229Term)	2	Morocco
		c.848 T > C (p.Val283Ala)/c.848 T > C (p.Val283Ala)	1	Spain
		c.848 T > C (p.Val283Ala)/c.996delT (p.Ala333Profs*20)	1	Venezuela
		c.761G > A (p.Gly254Asp)/c.761G > A (p.Gly254Asp)	1	Spain
		c.520G > A (p.Val174Met)/c.1097G > A;c.1844G > A (p.Arg366His;p.Arg615Gln)	1	Spain
		c.199A > T (p.Lys67Term)/c.1121A > C (p.His374Pro)	1	Germany‐Spain
		c.138 + 2 T > C /c.1366C > T (p.Arg456Cys)	1	Spain
		c.1367G > A (p.Arg456His)/c.1678 + 19_1678 + 31del13	1	Spain
		c.1174G > C (p.Val392Leu)/c.1752‐2_1755del6	1	Spain
		c.1077G > A (p.Ala359Ala)/c.683 T > C (p.Ile228Thr)	1	Italy‐Spain
LCHADD (2)	HADHA	c.1528G > C (p.Glu510Gln)/c.1915_1918delTATC (p.Tyr639Argfs*4)	1	Spain
		c.453 + 1G > A (p.Met106fs)/c.453 + 1G > A (p.Met106fs)	1	Ecuador
SPCD (11)	SLC22A5	c.845G > A (p.Arg282Gln) /c.845G > A (p.Arg282Gln)	1	Ecuador
		c.845G > A (p.Arg282Gln) /c.1392_1409del18ins2 (p.Val465Thrfs*29)	1	Bolivia/Ecuador
		c.806delT (p.Leu269Hisfs*27)/c.845G > A (p.Arg282Gln)	1	Spain‐Argentina
		c.760C > T (p.Arg254Ter)/C.1400C > G (p.Ser467Cys)	1	China
		c.743 T > C (p.Leu248Pro)/c.806delT (p.Leu269Hisfs*27)	1	Spain
		c.680G > A (p.Arg227His)/c.824 + 1G > T	1	Italy‐Spain
		c.447C > G (p.Phe149Leu)/c.680G > A (Arg227His)	1	Spain
		c.419G > A (p.Trp140Ter)/c.845G > A (p.Arg282Gln)	1	Peru
		c.364G > T (p.Asp122Tyr)/c.791C > G (Thr264Arg)	1	Spain
		c.1345 T > G (p.Tyr449Asp)/c.1072 T > A (p.Tyr358Asn)	1	Dominican Republic
		c.646G > C (p.Val216Leu)/c.646G > C (p.Val216Leu)	1	Morocco
CPT II (2)	CPT2	c.1547 T > C (p.Phe516Ser)/c.122_130del9(p.Pro41_Met43del)	1	Colombia‐Spain
		c.587C > T (p.Pro196Leu)/c.587C > T (p.Pro196Leu)	1	Spain
MADD (1)	ETFB	c.145G > C (p.Ala49Pro) /c.343_345delGAG (p.Glu115del)	1	Spain
CPT I (1)	CPT1A	c.2125G > A (p.Gly709Arg) /c.1948G > A (p.Gly650Ser)	1	Spain
MMAHC (7)	MMACHC	c.271dupA (p.Arg91Lysfs*14)/c.271dupA (p.Arg91Lysfs*14)	4	Spain (2) Morocco (2)
		c.271dupA (p.Arg91Lysfs*14)/c.440G > A (Gly147Asp)	1	Spain
		c.271dupA (p.Arg91Lysfs*14)/c.464G > A (p.Gly155Glu)	1	Spain
	MMADHC	c.748C > T (p.Arg250Ter)/c.748C > T (p.Arg250Ter)	1	Spain
MMA (4)	MMUT	c.322C > T (p.Arg108Cys)/c.2026G > A (p.Ala676Thr)	1	Spain
		c.655A > T (p.Asn219Tyr)/c.2206C > T (p.Leu736Phe)	1	Bulgaria
	MMAB	c.220G > T (p.Glu74Ter)/c.548A > T (p.His183Leu)	1	Spain
		c.662 T > G (p.Phe221Cys)/c.569G > A (p.Arg190His)	1	Spain

Abbreviations: BCAT‐2, branched‐chain amino acid transferase 2 deficiency; CPT‐I, carnitine palmitoyltransferase type 1 deficiency; CPT‐II, carnitine palmitoyltransferase type 2 deficiency; CTLN1, citrullinemia type 1; GA‐1, glutaric aciduria type 1; HC, homocystinuria; HMG‐CLD, 3‐hydroxy‐3‐methylglutaryl‐CoA lyase deficiency; HPA, hyperphenylalaninemia; IEM, inborn error of metabolism; LCHADD, long‐chain 3‐hydroxyacyl‐CoA dehydrogenase deficiency; MADD, multiple acyl‐coA dehydrogenation deficiency; MAT, hypermethioninemia; MCADD, medium‐chain acyl‐CoA dehydrogenase deficiency; MCG‐3:3‐methylcrotonyl‐CoA carboxylase deficiency; MMA, methylmalonic acidemia; MMAHC, methylmalonic acidemia with homocystinuria; MSUD, maple syrup urinary disease; OTC, ornithine transcarbamylase deficiency; PA, propionic acidemia; PKU, phenylketonuria; SPCD, systemic primary carnitine deficiencies; TYRSN‐1, tyrosinemia type I; TYRSN‐3, tyrosinemia type 3; VLCAD, very long‐chain acyl‐CoA dehydrogenase deficiency.

## 4 DISCUSSION

In our population, 26 different IEM have been detected, whereas pathogenic variants have been identified in 32 different genes.

An IEM was diagnosed in 1/2670 newborns, a remarkably similar rate was found in other studies performed in our Country (in the region of Galicia: 1/2060, excluding benign HPA^17^; in the region of Aragon^:^ 1/2573^18^; in the region of Murcia: 1/1884, including Cystic Fibrosis^19^). This rate is also similar in other Western European Countries: Portugal: 1/2396,^20^ Germany: 1/2712,^21^ or Italy: 1/2000^22^). As in other programs, the conditions with the highest incidence were HPA (1/6587) and MCAD deficiencies (1/13787).^9^, ^23^ A recent systematic review and meta‐analysis reported a global worldwide birth prevalence of PAH deficiency of 1/15625, being higher in Europe (1/8771).^24^

The positive predictive value was lower than in other reports in our Country, like in Galicia (76.11%),^17^ where they performed second tier tests and collected blood and urine samples simultaneously of all newborns; but comparable to other programs, where second tier tests were also not requested (20%).^19^ Nevertheless, the ENBS allowed the diagnosis of other entities different from IEM as vitamin B12 deficiencies, or different maternal diagnoses, which have not been included in this report. These conditions have already been reported in other programs.^25^, ^26^

Most of the cases benefited from presymptomatic diagnosis. Only 19 patients (8.5%) displayed symptoms before the result of the NBS was available. However, for some fatty acid oxidations disorders, organic acidemias and urea cycle disorders, at least half of the patients presented with clinical symptoms (LCHADD: 1/2, CPT‐II: 1/2, PA: 5/6, MMAHC: 3/7, MSUD: 2/4, OTC deficiency 2/2). Only one patient with MCADD, who presented with remarkable high octanoylcarnitine (C8) levels, displayed clinical symptoms (hypoglycemia and hypotonia). He harbored a homozygous pathogenic variant c.985A > G in the *ACADM* gene, which has been associated with more severe cases and higher levels of neonatal C8 and urinary acylglycines.^27^, ^28^ One of the patients diagnosed with LCHADD had a very early and fatal onset. This disease usually displays immediate complications, but recent data have shown that outcomes can be favorable if early diagnosis and strict dietary regime are initiated.^29^, ^30^ A similar scenario is evident in CPT‐II deficiency, with frequent sudden death or severe metabolic decompensation.^31^

For PA and MMA (including MMAHC) several cases presented with neurological deterioration in the first‐second weeks of life. It is well known that their clinical course generally starts with an acute metabolic decompensation in the neonatal period,^32^ that frequently leads to irreversible neurological damage.^33^ However, as in our cases, most of the patients are already symptomatic before NBS results can be available.^34^, ^35^ Furthermore, even when early diagnosis and optimal metabolic control are achieved, disease progression occurs.^36^ It has also been postulated that NBS does not have an effect on diagnosis of PA as it is hardly ever asymptomatic.^37^ However, as last guidelines stated,^38^ we consider necessary to establish a prompt diagnosis in children with suggestive clinical signs and symptoms. On the other hand, for GA‐1, as in our series, NBS usually allows the presymptomatic diagnosis, and it has been proven to be effective in preventing the progressive neurological deterioration.^39^, ^40^ Two of the three patients diagnosed with MSUD were symptomatic, and both have displayed neurological impairment in the follow‐up. Indeed, detection of this disorder before the occurrence of severe symptoms^41^ has been reported and NBS has demonstrated to avoid neurological deterioration and to clearly improve prognosis of MSUD patients.^42^, ^43^ Diagnosis of OTC by NBS is feasible as low blood citrulline levels can be detected in this condition.^44^ Notwithstanding, its inclusion in NBS programs remains controversial as hypocitrullinemia may not be present, especially in late onset forms.^45^ Based on our data, OTC should be evaluated and ruled‐out when low citrulline levels are detected to diagnose severe cases which can be potentially fatal, as our patient who died on the third day of life. Finally, one patient with Tyr‐I also displayed clinical symptoms with an acute liver failure. For this IEM, early diagnosis and treatment has also proven to have a benefit in the natural history although patients seem to remain with several neurological disturbances.^46^, ^47^

Mean time to clinical referral varied notably among the different IEMs. As shown in Table 1, time was longer for conditions considered “mild” as benign HPA, MCG‐3, or SPCD, where samples are frequently repeated before referral. The routine screening of MCG‐3 condition is controversial. In general, authors consider that longer investigations and follow‐up are needed to establish its indication.^48^ Besides, based on our data, we consider mandatory the accurate evaluation of cases with abnormal C5OH, as our patient with HMG‐CoA lyase deficiency presented with similar C5OH values as those with MCG‐3, without other biochemical abnormalities. For this condition, novel biomarkers have recently been identified^49^; organic acid analysis in urine is necessary as the second tier‐test for confirmation of this disease. Detection of HMG‐CoA lyase deficiency is essential as about half of the patients become symptomatic within the neonatal period.^50^

The death rate in our population was 1.3% (3/222), a bit lower than observed in other series.^17^, ^51^ Early mortality data for PA, LCHADD and OTC was 17, 50, and 50%, respectively. It is essential to report also longitudinal outcome data.

In our program, genetic diagnosis not only has confirmed all the biochemical cases (except for two) but also has allowed to detect novel IEM, as HPA due to DNAJC12 pathogenic variants and BCAT‐2 deficiency. These new two entities present with a heterogeneous clinical spectrum,^52^, ^53^ and description of the cases with long‐term studies are essential to better understand the natural history of these pathologies. Other recent studies have also established that molecular analyses can increase the number of pathologies from NBS,^54^ and some authors even postulate that whole exome sequencing could be considered as a follow‐up test for MS/MS positive individuals, offering an early and accurate definitive diagnosis.^55^, ^56^

For HPA, as it has been previously described,^57^ molecular characterization was heterogeneous. We have identified close to 60 different pathogenic variants, all of them with a low prevalence. More than 950 variants in the PAH have been identified, being the most one, the missense change c.1222C > T (23% of all variants).^58^ However, in our patients, it was only found in four alleles. In our series, the most prevalent variant was the c.1208C > T associated with benign HPA, and the second one the variant c.842C > T, which was not identified in a previous study conducted in our Country.^59^

Concerning MCADD, the most common variant was c.985A > G, found in 59 of 86 alleles (66%). In Galicia, the proportion was higher with a prevalence of 86% (78 out of 90 alleles).^60^ In fact, this variant is the most frequently reported in literature and occurs at a frequency up to 90% of disease alleles in symptomatic MCADD patients of European origin.^27^ However, as NBS has enabled the detection of patients with milder phenotype, the molecular heterogeneity of this defect has increased. Consequently, patients diagnosed within the screening generally show a lower proportion (30–71%) of that common variant.^27^, ^61^ In addition, a good correlation between genotype and enzyme function has recently been demonstrated.^62^

In conclusion, in 9 years, 222 IEM have been detected with a large clinical, biochemical, and molecular heterogeneity. Most of the cases benefited from presymptomatic diagnosis but with quite notable differences among the different disorders and 27 novel variants have been reported.

## CONFLICT OF INTEREST

The authors declare no potential conflict of interest.

## AUTHOR CONTRIBUTIONS

**Consuelo Pedrón‐Giner**: Had the original idea and contributed to planning the research design, methods, and preparation of manuscript. **Álvaro Martín‐Rivada** and **Laura Palomino Pérez**: Contributed to data acquisition, carrying out aspects of the methods and statistical analysis, and writing the draft of the manuscript. **Belén Pérez**: Performed, reviewed, and discussed data concerning molecular and genetic diagnosis. All authors have been involved in drafting the article and have expressed their agreement to submission.

## INFORMED CONSENT

All procedures followed were in accordance with the ethical standards of the responsible committee on human experimentation (institutional and national) and with the Helsinki Declaration of 1975, as revised in 2000. Informed consent was obtained from all patients for being included in the study.

## Supporting information


**Supplementary Material S1** Novel variants detected after the Implementation of Expanded Newborn Screening in Madrid.
dbSNP: Database for Single Nucleotide Polymorphisms
PhiloP
GVGD: Grantham Variation ‐ Grantham Deviation
SIFT: Scale‐invariant feature transform
CSVS: Collaborative Spanish Variant Server
MAF: Minimum allele frequency
GnomAD: Genome Aggregation Database
ACMG: American College of Medical Genetics

**Supplementary Material S2** Novel variants affecting intronic sites detected after the Implementation of Expanded Newborn Screening in Madrid.
SSF: Splicing Sequences Finder
MaxEnt: Maximum Entropy Modeling
NNSPLICE: Splice Site Prediction by Neural Network
CSVS: Collaborative Spanish Variant Server
MAF: Minimum allele frequency
GnomAD: Genome Aggregation Database
ACMG: American College of Medical Genetics

## Data Availability

Data archiving is not mandated but data will be made available on reasonable request.
