# Supplementary material for: Diagnosis of inborn errors of metabolism within the expanded newborn screening in the Madrid region
Source: JIMD Rep. 2022 Jan 27;63(2):146–61. doi: 10.1002/jmd2.12265 (PMC8898721; doi:10.1002/jmd2.12265)
Supplement: Supplementary file 1 — Supplementary Material S1 Novel variants detected after the Implementation of Expanded Newborn Screening in Madrid. dbSNP: Database for Single Nucleotide Polymorphisms PhiloP GVGD: Grantham Variation ‐ Grantham Deviation SIFT: Scale‐invariant feature transform CSVS: Collaborative Spanish Variant Server MAF: Minimum allele frequency GnomAD: Genome Aggregation Database ACMG: American College of Medical Genetics [file JMD2-63-146-s001.docx]

| **Table 4. Novel variants detected after the Implementation of Expanded Newborn Screening in Madrid.** | | | | | | | | | | | |  | |  |
| --- | --- | --- | --- | --- | --- | --- | --- | --- | --- | --- | --- | --- | --- | --- |
| **Gene Transcript** | VARIANT | dbSNP (151) | **Exon** | **Prediction (AlamutVisual®) v.2.14.0** | | | | | | | | **Allele Frecuency** | | **ACMG** |
|  |  |  |  | **PhiloP [-14.1;6.4]** | **GVGD v2007** | **Grantham dist [0-215]** | **SIFT  (v6.2.0)** | **Polyphen2 (v 2.2)[1-2]** | **Mutation Taster (v2013)** | **Provean (v4) Cutoff -2.5** | **Mutation Assessor (v4)** | **CSVS MAF** | **gnomAD (2.1)** |  |
| **ACADM NM_000016.5** | c.626C>T (p.Pro209Leu) Chr1(GRCh37):g.76211517C>T | rs1272058909 | 8 | 2.06 | C0  (GV: 228.00 - GD: 76.50) | 98 | Deleterious  (score: 0.02) | Benign  (score: 0.007) | Disease causing (prob: 1) | Damaging -6.8 | Low 1.445 | __ | 0.00040% | Likely pathogenic |
| **ACADVL NM_000018.3** | c.683T>C (p.Ile228Thr) Chr17(GRCh37):g.7125331T>C | __ | 8 | 4.64 | C25  (GV: 28.68 - GD: 69.84) | 89 | Deleterious (score: 0.01) | Probably damaging (score:1.000) | Disease causing (prob: 1) | Damaging -4.74 | Low 1.445 | __ | __ | Likely pathogenic |
| **ACADVL NM_000018.3** | c.1366C>T p.(Arg456Cys)  Chr17(GRCh37):g.7127320C>T | rs794727111 | 14 | 2.63 | C45  (GV: 28.82 - GD: 169.74) | 180 | Deleterious  (score: 0) | Probably damaging (score:0.999) | Disease causing (prob: 1) | Damaging -7.93 | High 4.11 | __ | 0.0064% | Likely pathogenic |
| **BCKDHA  NM_000709.4** | c.743C>T (p.Ala248Val)  Chr19(GRCh37):g.41928165C>T | Rs887411374 | 6 | 5.45 | C0  (GV: 157.81-  GD: 0.00) | 64 | Tolerated  (score: 0.27) | Probably damaging (score:0.928) | Disease causing (prob: 1) | Damaging -3.89 | Medium 3.11 | __ | __ | Likely pathogenic |
| **BCKDHB  NM_183050.3** | c.604G>A (p.Ala202Thr)  Chr6(GRCh37):g.80878718G>A | rs369388961 | 5 | 5.37 | C0  (GV: 26.87 - GD: 37.18) | 58 | Tolerated  (score: 0.06) | Probably damaging (score:0.923) | Disease causing (prob: 1) | Damaging -3.26 | Medium 2.845 | __ | 0.00080% | Likely pathogenic |
| **CPT1A NM_001876.3** | c.1948G>A (p.Gly650Ser) Chr11(GRCh37):g.68529083C>T | rs760769734 | 16 | 4.48 | C0  (GV: 206.04 - GD: 29.56) | 56 | Deleterious  (score: 0) | Probably damaging (score:0.999) | Disease causing (prob: 1) | Damaging -5.56 | High 4.38 | __ | __ | Uncertain significance |
| **CPT1A NM_001876.3** | c.2125G>A (p.Gly709Arg) Chr11(GRCh37):g.68527710C>T | __ | 17 | 4.24 | C15  (GV: 206.04 - GD: 124.98) | 125 | Deleterious  (score: 0) | Probably damaging (score:0.999) | Disease causing (prob: 1) | Damaging -6.77 | High 4.055 | __ | __ | Uncertains significance |
| **CPT2 NM_000098.2** | c.587C>T (p.Pro196Leu) Chr1(GRCh37):g.53675933C>T | rs758823353 | 4 | 5.61 | C0  (GV: 208.63 - GD: 94.04) | 98 | Deleterious  (score: 0) | Probably damaging (score:0.991) | Disease causing (prob: 1) | Damaging -9.56 | High 3.515 | __ | __ | Uncertain significance |
| **GCDH NM_000159.3** | c.1210G>C (p.Ala404Pro)  Chr19(GRCh37):g.13008644G>C | __ | 11 | 3.03 | C0  (GV: 65.28 - GD: 26.78) | 27 | Tolerated  (score: 0.07) | Probably damaging (score:0.877) | Disease causing  (prob: 757) | Neutral -2.34 | Medium 3.035 | __ | __ | Likely pathogenic |
| **HMGCL NM_000191.2** | c.785G>A (p.Gly262Glu) Chr1(GRCh37):g.24130981C>T | __ | 8 | 5.94 | C65  (GV: 0.00 –  GD: 97.85) | 98 | Deleterious  (score: 0) | Probably damaging (score:1.000) | Disease causing (prob: 1) | Damaging -7.6 | High 3.795 | __ | __ | Likely pathogenic |
| **HPD NM_002150.2** | c.1118A>T (p.Glu373Val) Chr12(GRCh37):g.122277698T>A | __ | 17 | -0.04 | C0  (GV: 263.50 - GD: 0.00) | 121 | Tolerated  (score: 1) | Benign  (score: 0.004) | Disease causing (prob: 0.91) | Neutral 1.62 | __ | __ | __ | Likely  benign |
| **HPD NM_002150.2** | c.778G>A (p.Gly260Arg) Chr12(GRCh37):g.122284821C>T | rs755816788 | 14 | 6.02 | C65  (GV: 0.00 –  GD: 125.13) | 125 | Deleterious  (score: 0) | Probably damaging (score:0.994) | Disease causing (prob: 1) | Damaging -6.82 | __ | __ | 0.0020% | Uncertain  significance |
| **MMAB**  **NM_052845.4** | c.662T>G (p.Phe221Cys) Chr12(GRCh37):g.109994**924**A>C | __ | 9 | 2.95 | C65  (GV: 0.00 –  GD: 204.39) | 205 | Deleterious  (score: 0) | Probably damaging (score:1.000) | Disease causing (prob: 1) | Damaging -7.16 | High 4.745 |  | __ | Likely pathogenic |
| **MAT1A NM_000429.2** | c.770G>A (p.Gly257Glu) Chr10(GRCh37):g.82034954C>T | rs371264669 | 7 | 5.29 | C0  (GV: 109.55 - GD: 54.82) | 98 | Deleterious  (score: 0.04) | Probably damaging (score:0.979) | Disease causing (prob: 1) | Damaging -6.81 | High 3.755 | __ | __ | Uncertain significance |
| **MCCC1 NM_020166.4** | c.1970T>C (p. Ile657Thr) Chr3(GRCh37):g.182737925A>G | rs1194507396 | 17 | 3.84 | C15  (GV: 50.29 - GD: 69.84) | 89 | Deleterious  (score: 0.01) | Possibly damaging (score:0.864) | Disease causing (prob: 1) | Damaging -4.09 | High 4.01 | __ | __ | Likely pathogenic |
| **SLC22A5 NM_003060.3** | c.646G>C (p.Val216Leu) Chr5(GRCh38):g.132384295G>C | __ | 3 | 6.10 | C0  (GV: 28.68 - GD: 4.86) | 32 | Tolerated  (score: 0.06) | Possibly damaging (score:0.458) | Disease causing (prob: 1) | Damaging -7.6 | Medium 3.11 | __ | __ | Likely pathogenic |
| **SLC22A5 NM_003060.3** | c.743T>C (p.Leu248Pro) Chr5(GRCh37):g.131 721 110T>C | __ | 4 | 1.42 | C0  (GV: 137.69 - GD: 0.00) | 98 | Deleterious  (score: 0.05) | Possibly damaging (score:0.810) | Disease causing (prob: 1) | Damaging -5.11 | Medium 3.39 | __ | __ | Likely pathogenic |
| **PAH NM_000277.3** | c.506_508delGCCinsCCA (p.Arg169_His170delinsProAsn) Chr12(GRCh37):g.103260375_103260377delinsTGG | __ | 5 | __ | __ | __ | __ | __ | __ | __ | __ | __ | __ | Likely pathogenic |
| **ACADM NM_000016.5** | c.778_782delGAAAA (p.Glu260Cysfs*5) Chr1(GRCh37):g.76215173_76215177del | __ | 9 | __ | __ | __ | __ | __ | __ | __ | __ | __ | __ | Pathogenic |
| **ACADVL NM_000018.3** | c.1678+19_1678+31delATTCCGCCTCC Chr17(GRCh37):g.7127890_7127902del | __ | Intron 17 | __ | __ | __ | __ | __ | __ | __ | __ | __ | __ | Uncertain significance |
| **CPT2 NM_000098.2** | c.122_130del9 (p.Pro41_Met43del) Chr1(GRCh37):g.53662737_53662745del | __ | 1 | __ | __ | __ | __ | __ | __ | __ | __ | __ | __ | Likely pathogenic |
