# Supplementary material for: Diagnosis of inborn errors of metabolism within the expanded newborn screening in the Madrid region
Source: JIMD Rep. 2022 Jan 27;63(2):146–61. doi: 10.1002/jmd2.12265 (PMC8898721; doi:10.1002/jmd2.12265)
Supplement: Supplementary file 2 — Supplementary Material S2 Novel variants affecting intronic sites detected after the Implementation of Expanded Newborn Screening in Madrid. SSF: Splicing Sequences Finder MaxEnt: Maximum Entropy Modeling NNSPLICE: Splice Site Prediction by Neural Network CSVS: Collaborative Spanish Variant Server MAF: Minimum allele frequency GnomAD: Genome Aggregation Database ACMG: American College of Medical Genetics [file JMD2-63-146-s002.docx]

| **Gene Transcript** | **VARIANT** | dbSNP (151) | Intron | **splicing** | | | | **Allele frequency** | | **ACMG** |  |  |  |  |
| --- | --- | --- | --- | --- | --- | --- | --- | --- | --- | --- | --- | --- | --- | --- |
|  |  |  |  | **SSF [0-100]** | **MaxEnt  [0-16]** | **NNSPLICE  [0-1]** | **GeneSplicer [0-15]** | **CSVS   MAF** | **gnomAD (2.1)** |  |  |  |  |  |
| **ACADVL NM_000018.3** | **c.138+2T>C (p.?) Chr17(GRCh37):g.7123518T>C** | rs1057516817 | intron 2 | = 100.00 | 10.86 ⇒ — | 1.00 ⇒ — | 15.18 ⇒ — | __ | 0.00078% | Pathogenic |  |  |  |  |
| **DNAJC12 NM_021800.2** | **c.502+1G>C (p.?) Chr10(GRCh37):g.69565340C>G** | __ | intron 4 | 100.00 ⇒ — | 10.86 ⇒ — | 1.00 ⇒ — | 6.93 ⇒ — | __ | __ | Pathogenic |  |  |  |  |
| **DNAJC12 NM_021800.2** | **c.298-2A>C (p.?) Chr10(GRCh37):g.69565547T>G** | __ | intron 3 | 70.70 ⇒ — | 4.70 ⇒ — |  |  | __ | __ | Pathogenic |  |  |  |  |
| **HADHA NM_000182.4** | **c.453+1G>A (p.?) Chr2(GRCh37):g.26457084C>T** | rs1057516417 | intron 5 |  | 7.13 ⇒ — |  | 1.81 ⇒ — | __ | __ | Pathogenic |  |  |  |  |
| **MCCC2 NM_022132.4** | **c.804-14T>A (p.?) Chr5(GRCh37):g.70930756T>A** | __ | intron 8 | 3.38 ⇒ — | 5.64 ⇒ 1.76 *(-68.8%)* | 0.90 ⇒ 0.73 *(-18.0%)* | 1.02 ⇒ — | __ | __ | Uncertain significance |  |  |  |  |
| **PAH NM_000277.3** | **c.441+87T>G (p.?) Chr12(GRCh37):g.103271153A>C** | rs1010831842 | intron 4 | — ⇒ 76.52 | — ⇒ 8.40 | — ⇒ 0.88 | — ⇒ 1.28 | __ | 0.0032% | Uncertain significance |  |  |  |  |

**Table 5. Novel variants affecting intronic sites detected after the Implementation of Expanded Newborn Screening in Madrid.**
